# Importance of whole genome sequencing for the assessment of outbreaks in diagnostic laboratories: analysis of a case series of invasive *Streptococcus pyogenes* infections

**DOI:** 10.1007/s10096-017-2905-z

**Published:** 2017-01-26

**Authors:** F. Tagini, B. Aubert, N. Troillet, T. Pillonel, G. Praz, P. A. Crisinel, G. Prod’hom, S. Asner, G. Greub

**Affiliations:** 1 Institute of Microbiology, Department of Laboratory, University of Lausanne & Lausanne University Hospital, Lausanne, Switzerland; 2 Unit of Pediatric Infectious Diseases and Vaccinology, Department of Pediatrics, Lausanne University Hospital, Lausanne, Switzerland; 3 Service of Infectious Diseases, Central Institute of the Valais Hospitals, Sion, Switzerland; 4 Service of Infectious Diseases, Department of Internal Medicine, Lausanne University Hospital, Lausanne, Switzerland

**Keywords:** Streptococcus pyogenes, Group a streptococcus, Whole genome sequencing, Outbreak, Emm-typing, MLST

## Abstract

Outbreaks of *Streptococcus pyogenes* hypervirulent clones are constant public health threats. In western Switzerland, an increase of severe cases of *S. pyogenes* invasive infections was observed between December 2015 and March 2016. Our aim was (i) to investigate these cases by the use of Whole Genome Sequencing (WGS) and (ii) to determine the specific virulome and resistome of each isolate in order to undertake adequate public health measures. Eleven *Streptococcus pyogenes* strains isolated from 11 patients with severe invasive infections between December 13, 2015 and March 12, 2016 were included in our study. Practically, *emm*-typing, MLST and WGS were used to investigate the relatedness between the isolates. The presence of virulence and antibiotic resistance genes as well as mutations in transcriptional regulators of virulence and in genes encoding for antibiotic targets were assessed. Three and two groups of isolates shared the same *emm*-type and ST type, respectively. Single Nucleotide Polymorphism (SNP) analysis revealed 14 to 32 SNPs between the strains of the same *emm*-type group, ruling out the possibility of a clonal outbreak. Mutations found in *covS* and *rocA* could partially explain an increased virulence. As these reassuring results were obtained in less than 10 days, no specific hospital hygiene and no dedicated public health measures had to be undertaken. WGS is a powerful technique to discriminate between closely related strains, excluding an outbreak in less than 10 days. Moreover, WGS provided extensive data on the virulome and resistome of all these strains.

**Electronic supplementary material:**

The online version of this article (doi:10.1007/s10096-017-2905-z) contains supplementary material, which is available to authorized users.

## Introduction

*Streptococcus pyogenes* (Group A beta-hemolytic Streptococcus, GAS) is a colonizer of the human oropharynx and skin that can cause very diverse clinical presentations ranging from common and limited diseases such as pharyngo-tonsillitis, impetigo, erysipelas or cellulitis to life-threatening invasive diseases such as necrotizing fasciitis, pneumonia or streptococcal toxic shock syndrome [1]. The burden of invasive GAS infections in western countries is significant with an average incidence of 2.45 cases per 100,000 person-year and a case fatality rate of 15% [2]. Outbreaks of hypervirulent clones have been reported [3–5]. Their dramatic spread remains a constant public health threat, which requires a quick assessment.

Typing of *S. pyogenes* strains was historically done using a serologic classification of the M protein described by Lancefield in 1928. Nowadays, the *emm* gene, encoding for the M protein, allows the typing of isolates using PCR and sequencing of the amplicons, but not to discriminate between two isolates of the same *emm*-type and subtype. The same limitation applies to other typing approaches such as (i) multilocus sequence typing (MLST), based on the amplification and sequencing of a few housekeeping genes [6] or (ii) pulsed-field gel electrophoresis (PFGE) of large genomic fragments, previously cut by restriction enzymes [7]. Whole genome sequencing (WGS) has recently entered diagnostic laboratories and becomes a very powerful tool that allows differentiation between isolates at the level of Single Nucleotide Polymorphism (SNP) as well as a thorough investigation of the presence of specific virulence and antibiotic resistance genes [8]. Furthermore, mutations in two-components systems, in transcriptional regulators or in regulatory proteins (e.g. *covR/covS (csrR/csrS)*, *ropB/rgg* or *rocA* respectively) have been shown to increase GAS virulence [9, 10], and the presence of such mutations may be investigated in order to get insights into the isolate’s propensity for invasion.

From January 2016 to March 2016, an increase of severe *Streptococcus pyogenes* infections was reported in Valais, a Swiss alpine canton with a population of 360,000 inhabitants. Indeed, six cases of GAS bacteremia were reported from January 1, 2016 to March 20, 2016, whereas only six, ten and eight GAS bacteremia were reported in 12 months in 2013, 2014 and 2015, respectively. Thus, we investigated a potential outbreak due to a hypervirulent *S. pyogenes* clone using genomics and analyzed the virulome and resistome of the isolated strains.

## Methods

### Clinical isolates and clinical data

We investigated *Streptococcus pyogenes* strains isolated between December 13, 2015 and March 12, 2016 from inpatients from Valais, who were included in this study as well as all the isolates obtained from normally sterile sites or from normally non-sterile sites providing a severe clinical presentation defined as sepsis, septic shock/toxic shock syndrome, meningitis, pneumonia or necrotizing fasciitis [11, 12]. Clinical data were retrieved from the patients’ electronic charts.

### DNA extraction and whole genome sequencing

Genomic DNA extraction and purification were done using the protocol for Gram-positive bacteria with the Wizard Genomic DNA Purification Kit (Promega, ref. A1120). Genomic libraries were made using Nextera XT library kit (Illumina). One hundred fifty base pairs (bp) paired-end sequencing was performed using a MiSeq sequencer (Illumina, San Diego CA).

### Assembly and annotation

Reads quality was assessed using FastQC version 0.11.5 (Andrews S. (2010), available online at: http://www.bioinformatics.babraham.ac.uk/projects/fastqc). Trimmomatic 0.35 was used to filter low quality reads and reads shorter than 150 bp [13]. Assemblies were done with SPAdes genome assembler version 3.6.2 using k-mer sizes ranging from 43 to 127 bases [14]. Quast version 3.1 [15] was used to select the best assembly based on lowest number of contigs and best N50. Contigs shorter than 1000 bp and low k-mer coverage contigs (<2x) were discarded from the assemblies. The remaining contigs were reordered based on the RefSeq reference genome (strain SF370). Annotation was performed using RAST version 2.0 [16].

### *emm* typing and multilocus sequence typing

*emm*-types and subtypes were determined by submitting *emm* gene sequences to the website of the Centers for Disease Control and Prevention, *Streptococcus* laboratory (http://www2a.cdc.gov/ncidod/biotech/strepblast.asp). Genome assemblies were submitted online to the pubMLST (http://pubmlst.org) database in order to assess their MLST types.

### Core genomes alignment and mapping — whole genome alignments

The assemblies were aligned with the 51 complete genomes available on NCBI (Table S1) using Parsnp (v1.2) [17]. FigTree version 1.4.2 (http://tree.bio.ed.ac.uk/software/figtree/) was used to edit the phylogenic tree based on the core-genome alignment. In order to detect the genetic variants, the trimmed reads were mapped against the core-genome of a closely related reference (based on Parsnp results) using snippy version 3.0 [18].

The assemblies of closely related isolates were aligned with Mauve version 2.4.0 [19] to assess genetic gains and losses. We looked manually at the annotation with Artemis 16.0.0 [20] to identify the gained or lost coding sequences.

### Virulence factors

Virulence factors were identified by BLASTing the curated Virulence Factors Database (VFDB) [21] against the assemblies (cut-offs: e-value < 10^−5^, amino acid identity > 90%). Variants in the two-component regulatory system *covR/covS* and the transcriptional regulators *ropB* and *rocA* were identified by mapping trimmed reads against a reference genome (strain SF370) using snippy version 3.0 [18].

### Hyaluronic acid assay

Hyaluronic acid quantification was performed in two independent experiments. Isolates were grown in THB medium until mid-logarithmic phase, as described in Hollands et al. [22]. Five milliliters of the culture (OD_600_, 0,4) were centrifuged at 5000 *g* for 10 min. Then, supernatants were discarded and pellets of bacteria were resuspended into 500 μl of deionized water. Serial dilutions of bacterial suspension were plated on blood agar growth medium and incubated to calibrate the suspension in colony-forming unit (CFU) per ml. Four hundred microliters were mixed with 1 ml of chloroform, 1-millimeter beads and mechanically shaken with a Precellys Evolution Homogenizer (Bertin Instruments, Montigny-le-Bretonneux), three times for 30 sec at 6800 rpm. Tubes were then centrifugated at 13000 *g* for 10 min. Hyaluronic acid concentration in the aqueous phase was determined using Corgenix Hyaluronic Acid kit according to manufacturer’s instructions.

### Resistome and antibiotic susceptibility testing

Antibiotic resistance genes were identified using Resfinder version 2.1 with a threshold of 98% identity and 60% query coverage [23]. A manual search for known mutations in *parC*, *gyrA*, *folA* and *folP* was performed. Minimal inhibitory concentration for each isolate was determined using VITEK® 2 AST Cards (Biomérieux, France).

## Results

### Isolates and clinical presentations

Eleven *S. pyogenes* isolates were included in this study. Five out of six patients with complicated pneumonia were pediatric patients, whereas skin and soft tissues as well as joint infections were seen only in adult patients (Table 1). A report could be sent after less than 10 days after the reception of the strains in our laboratory.

**Table 1:** List of the isolates and the clinical presentations

Identifier	Gender	Age (years)	Sample	Primary diagnosis	Secondary diagnosis	Outcome
ISR1	F	1	Blood culture	Complicated pneumonia with bilateral empyemas and TSS	RSV Bronchiolitis	Good
ISR2	M	1	Pleural fluid	Complicated pneumonia with parapneumonic effusion	RSV infection	Good
ISR3	M	4	Blood culture	Complicated pneumonia with parapneumonic effusion and TSS	Influenza A infection	Good
ISR4	M	6	Pharyngeal swab	Oto-mastoiditis with subsequent meningitis and TSS	Influenza B infection	Good
ISR5	F	47	Blood culture	Pneumonia complicated by ARDS	–	Good
ISR6	F	1	Post-mortem, pleural fluid	Complicated pneumonia with parapneumonic effusion and TSS	–	Death
ISR7	M	49	Blood culture	Skin and soft tissues infection (cellulitis)	–	Good
ISR8	M	79	Blood culture	Skin and soft tissues infection (cellulitis) complicated by deep venous thrombosis and acute renal failure	–	Good
ISR9	F	58	Blood culture	Septic arthritis	–	Good
ISR10	F	76	Blood culture	Skin and soft tissues infection complicated by TSS	–	Good
ISR11	F	15	Blood culture	Complicated pneumonia with parapneumonic effusion	–	Good

*F* female, *M* male, *ARDS* acute respiratory distress syndrome, *TSS* toxic shock syndrome, *RSV* respiratory syncytial virus

### *emm*-type and MLST

Eight different *emm*-types were detected (Table 2). Three *emm*-types were recovered twice among the isolates: *emm*1, *emm*22 and *emm*28. Multilocus sequence typing was more discriminant and allowed the identification of nine different ST-types (Table 2). Eight ST-types were detected with all the loci matching exactly against the pubMLST database. ISR2 bore a new allele of *murI* (110), which was submitted to the database as well as its new ST-type. In summary, *emm*-typing and MLST did not allow discrimination between three and two pairs of strains, respectively.

**Table 2:** *emm*-types, *emm*-clusters and multi-locus sequence typing (ST types and the allelic profiles corresponding to the 7 loci)

Isolates	*emm*-type (.subtype)	*emm*-cluster	ST	*gki*	*gtr*	*murI*	*mutS*	*recP*	*xpt*	*yqiL*
ISR3	*emm*1.0	A-C3	28	4	3	4	4	4	2	4
ISR4	*emm*1.0	A-C3	28	4	3	4	4	4	2	4
ISR7	*emm*22	E4	46	9	8	1	1	1	3	4
ISR8	*emm*22	E4	46	9	8	1	1	1	3	4
ISR9	*emm*28.0	E4	458	11	77	14	5	9	17	19
ISR11	*emm*28.0	E4	52	11	6	14	5	9	17	19
ISR1	*emm*3.1	A-C5	15	2	6	8	5	2	3	2
ISR2	*emm*75.0	E6	880	11	2	110	3	50	8	7
ISR5	*emm*73.0	E4	331	43	2	2	47	1	3	4
ISR6	*emm*5.23	Y^a^	99	33	30	7	5	5	26	3
ISR10	*emm*89.0	E4	101	16	2	8	3	1	13	3

^a^Single protein *emm*-cluster clade Y

### Core-genome alignment and mapping

**Fig. 1:**
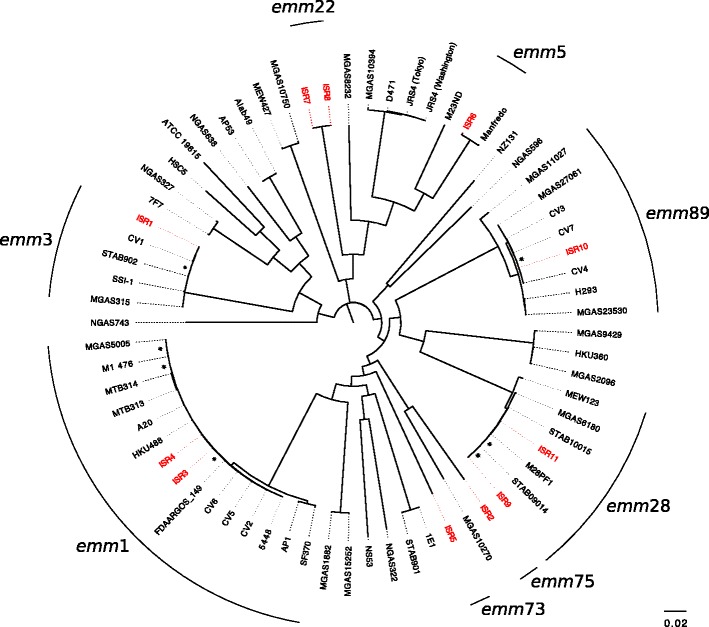
Phylogenetic representation of the sequenced strains during our study and the complete GAS genomes publicly available on NCBI. The phylogenetic tree was made using Parsnp and is based on the core genome alignment of our 11 isolates (in *red*), the currently 51 complete genomes available on NCBI (Table S1) and seven other genomes of *S. pyogenes* recently sequenced in our institute (from CV1 to CV7, taking part in another study). The tree was rooted at midpoint. *Stars* indicate bootstraps below 0.9

Genome assembly sizes ranged from 1,689,494 to 1,868,156 bp with a mean GC content of 38.35% (assemblies statistics are provided in supplementary materials, Table S2). The core genome size was 1,445,210 bp (see Parsnp results, Fig. 1). SNPs quantification in the core genomes of the closest isolates (corresponding to the same *emm*-types) revealed for *emm*1 isolates (ISR3 and ISR4), *emm*22 isolates (ISR7 and ISR8) and *emm*28 isolates (ISR9 and ISR11), 32, 14 and 28 SNPs of difference in their core genomes, respectively.

To look for additional differences (i.e. large insertions and deletions not detected by mapping) between the genomes of the closest isolates, we aligned the complete assemblies with progressive Mauve. ISR3 and ISR4 did not show any noticeable differences. ISR8 held 2 phage regions of 13.2 Kb and 14.6 Kb, the latest bearing *speK* gene whereas ISR7 had a 10.0 Kb phage region not retrieved in ISR8. ISR11 bore a genomic island of 24.7 Kb with a high content in hypothetical protein CDSs that was not present in ISR9.

### Virulence factors and regulation of virulence

Twelve to 24 virulence factors encoding genes were identified per isolate (Table 3). The *has*-operon was found in all but three isolates (ISR7, ISR8 and ISR10), which only bore the *hasC* gene. ISR9 and ISR11 presented a 1 bp-insertion responsible for a frame-shift and premature truncation of the *hasA* product at amino acid 72. All the isolates had two to five genes encoding for superantigens (mean = 4).

**Table 3:** Virulence genes detected by BLASTing the Virulence Factor Database (VFDB) on the assemblies

*emm1*		*emm22*		*emm28*		*emm3*	*emm75*	*emm73*	*emm5*	*emm89*
ISR3	ISR4	ISR7	ISR8	ISR9	ISR11	ISR1	ISR2	ISR5	ISR6	ISR10
*cpa*	*cpa*	*fbaA*	*fbaA*	*fbaA*	*fbaA*	*cpa*	*cpa*	*fbaA*	*fbaB*	*fbaA*
*emm*	*emm*	*fbp54*	*fbp54*	*fbp54*	*fbp54*	*emm*	*emm*	*fbp54*	*fbp54*	*fbaB*
*fbaA*	*fbaA*	*grab*	*grab*	***hasA***	***hasA***	*fbaB*	*fbaB*	***hasA***	***hasA***	*fbp54*
*fbp54*	*fbp54*	***hasC***	***hasC***	***hasB***	***hasB***	*fbp54*	*fbp54*	***hasB***	***hasB***	***hasC***
*fctA*	*fctA*	*hylP*	*hylP*	***hasC***	***hasC***	*grab*	*grab*	***hasC***	***hasC***	*lmb*
*fctB*	*fctB*	*ideS/mac*	*ideS/mac*	*lmb*	*lmb*	***hasA***	***hasA***	*lmb*	*lmb*	*mf/spd*
*grab*	*grab*	*lmb*	*lmb*	*mf/spd*	*mf/spd*	***hasB***	***hasB***	*mf/spd*	*mf/spd*	*scpA*
***hasA***	***hasA***	*mf/spd*	*mf/spd*	*mf2*	*mf2*	***hasC***	***hasC***	*mf2*	*mf2*	*scpB*
***hasB***	***hasB***	*mf2*	*mf2*	*scpA*	*scpA*	*hylP*	*hylP*	*scpA*	*mf3*	*slo*
***hasC***	***hasC***	*scpA*	*scpA*	*scpB*	*scpB*	*ideS/mac*	*ideS/mac*	*slo*	*scpA*	*smeZ*
*ideS/mac*	*ideS/mac*	*scpB*	*scpB*	*slo*	*slo*	*lmb*	*lmb*	*smeZ*	*scpB*	*speB*
*lepA*	*lepA*	*slo*	*slo*	*smeZ*	*smeZ*	*mf/spd*	*mf/spd*	*speB*	*slo*	*speG*
*lmb*	*lmb*	*smeZ*	*smeZ*	*speB*	*speB*	*scpA*	*scpA*	*speC*	*smeZ*	
*mf/spd*	*mf/spd*	*speB*	*speB*	*speC*	*speC*	*ska*	*ska*	*speG*	*speB*	
*mf3*	*mf3*	*speC*	*speC*	*speG*	*speG*	*slo*	*slo*	*speH*	*speC*	
*scpA*	*scpA*	*speG*	*speG*	*speJ*	*speJ*	*smeZ*	*smeZ*	*speI*	*speG*	
*ska*	*ska*	*ssa*	*speK*			*speB*	*speA*			
*slo*	*slo*		*ssa*			*speG*	*speB*			
*smeZ*	*smeZ*					*speK*	*speG*			
*speA*	*speA*					*ssa*	*speK*			
*speB*	*speB*						*ssa*			
*speG*	*speG*									
*speJ*	*speJ*									
*srtC1*	*srtC1*									

*Has* operon genes are shown in *bold* and superantigens are *underlined*. Of notes, *speB* is not considered as a superantigen and appears in *black*. For gene descriptions, see online supplementary materials, Table S4

**Fig. 2:**
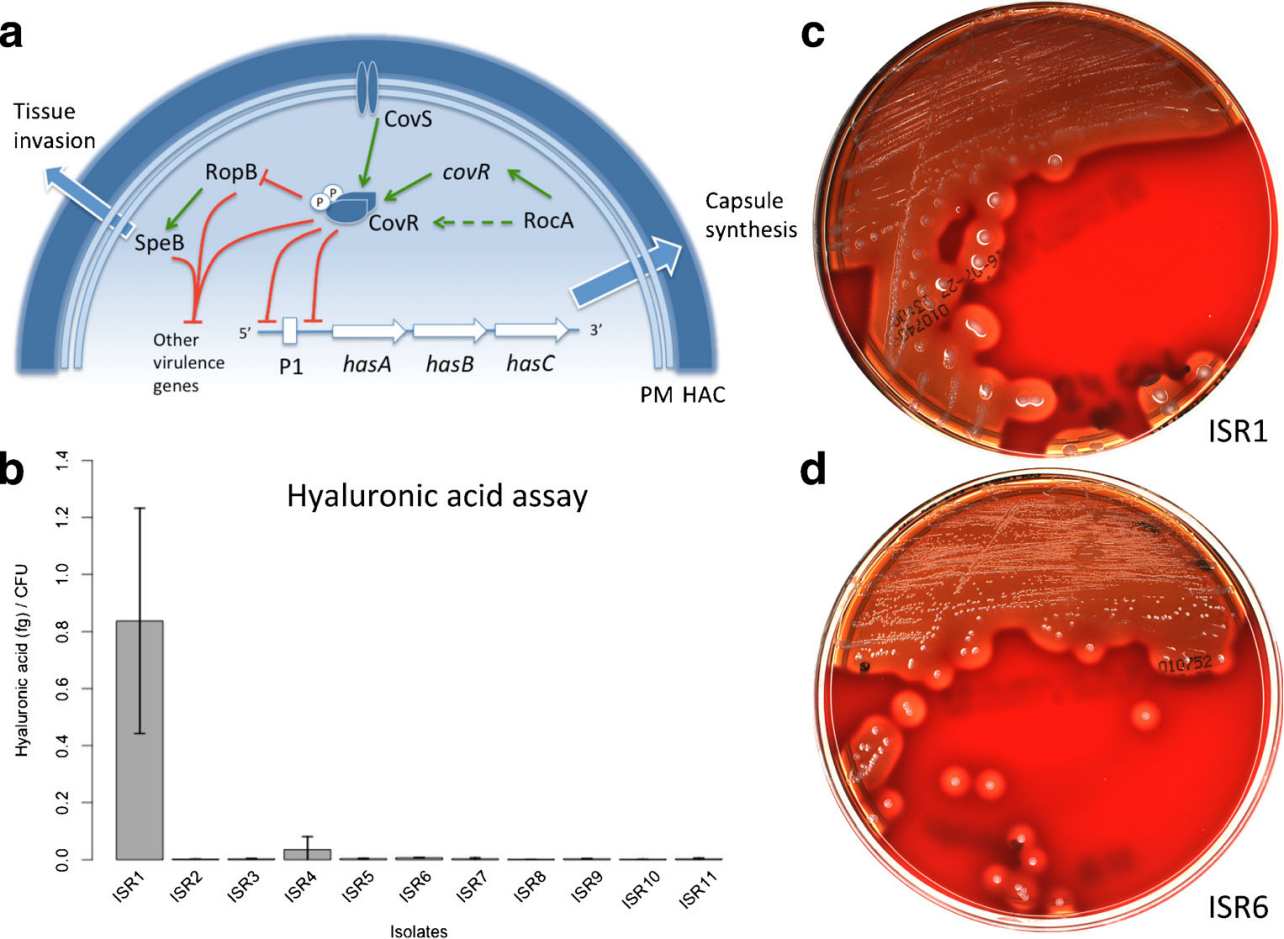
**a** Simplified scheme showing the best-known transcriptional regulators of virulence in *Streptococcus pyogenes* missense and non-sense mutations in *covR* and *covS* can increase *Streptococcus pyogenes* virulence by relieving CovR downregulation of many virulence genes such as the *has*-operon involved in hyaluronic acid synthesis [9]. RocA positively regulates *covR* transcription and possibly directly phosphorylates CovR [10]. Mutations resulting in premature truncation of RocA were shown to increase hyaluronic acid production and virulence. Mutations in *ropB*, a negative transcriptional regulator of many virulence genes, have been linked to more invasive phenotypes [24]. This regulator is also necessary for the expression of *speB*, a gene encoding for a cysteine protease degrading many *Streptococcus pyogenes* virulence factors and whose expression is inversely proportional to virulence, though *speB* is also known to be a major virulence factor involved in tissue invasion and in the pathogenesis of necrotizing fasciitis [9]. *PM* plasma membrane, *HAC* hyaluronic acid capsule, *P1* promoter region 1 of the *has* operon. **b** Enzyme-linked binding protein assay ISR1 (*emm*3.1) and ISR4 (*emm*1.0) produced a higher amount of hyaluronic acid. **c**, **d** Blood agar plates showing a mucoid phenotype (ISR1) and a non-mucoid phenotype (ISR6)

We found two mutations in *covS* and one in *rocA* that are associated with an increased virulence [24, 25] (Fig. 2a). ISR9 exhibited a non-synonymous SNP in *covS* responsible for an amino acid replacement; E226G and ISR11 had a single nucleotide deletion at position 528 responsible for a premature truncation of CovS at amino acid 180. These two *covS* mutations were already described by Ikebe et al. in an invasive *emm*28 (NIH35) and in an invasive *emm*3 isolate (NIH453), respectively [24]. ISR1 (*emm*3) showed a known deletion in *rocA* causing a premature truncation and a loss of function of the product, as described by Lynskey et al. [25]. The rest of the variants found in *covR*, *covS*, *ropB* and *rocA* (supplementary materials, Table S3) have not been linked to more invasive phenotypes yet. They seem to be *emm*-type specific and can also be found in strains recovered from non-invasive infections (data not shown).

### Hyaluronic acid assay

ISR1, which was the only isolate exhibiting a frank mucoid phenotype on agar plates (Fig. 2c), showed the highest production of hyaluronic acid of the assay, which is congruent with previous findings [25]. ISR4 produced a larger amount of hyaluronic acid than the other isolates (Fig. 2b) but does not exhibit any known mutations in the transcriptional regulator genes analyzed.

### Resistance genes

Two resistance genes were detected with ResFinder in the genome of the isolates. *erm(B)*, a chromosomal gene involved in antibiotic resistance against macrolides and lincosamides, was detected in the ISR11 genome. *tet(M)*, a tetracycline resistance gene, was found in ISR6, ISR7 and ISR8 genomes. Finally, ISR7 and ISR8 exhibited a non-synonymous mutation in *parC* (S79F), a gene involved in fluoroquinolone resistance [26].

### Antibiotic susceptibility test

Congruent with the genomic findings, ISR11 was resistant to clindamycin (MIC > 1) and erythromycin (MIC > 8) and ISR6, ISR7 and ISR8 displayed various levels of tetracycline resistance (MIC: 4, MIC > =16 and MIC > =16, respectively). ISR7 and ISR8 were also resistant to levofloxacin (MIC: 4), as expected from their mutations located in the quinolone resistance-determining region of *parC*. Interestingly, ISR1 presented an intermediate sensitivity to trimethoprim-sulfamethoxazole (MIC: 40).

## Discussion

An outbreak resulting from a hypervirulent clone was suspected due to the observed increased number of severe invasive GAS infections in western Switzerland over a 3-month period. In order to precisely and rapidly address this concern, we used whole genome sequencing (WGS) and we could rule out a clonal outbreak by comparing all the sequenced strains and by detecting the identification of 14 to 32 core-genome SNPs in the three pairs of closely related strains. This number of SNPs was much higher between the Swiss isolates taken less than 3 months apart compared to the 0–4 SNPs difference reported by Engelthaler et al. [4] when comparing clonal isolates drawn during a similar 3-month period. Moreover, Beres et al. [27] described a mean rate of 1.7 SNP/strain/year in the core genome, dating divergence of our closest isolates at about 8 years before sampling. We are confident that even the isolates bearing only 14 SNPs of difference in their core genome (ISR7 & ISR8) did not come from the same clone because they also had variations in their accessory genomes with different phage regions. Thus, this study showed the high discriminatory power of WGS, its possible application in clinical microbiology and emphasized the limitation of *emm*-typing and MLST, which did not allow differentiation of closely related strains of *S. pyogenes*. The use of WGS enabled rapid (<10 days) exclusion of a clonal outbreak, which reassured clinicians and public health authorities. This short time-to-result period had a positive impact on sparing hospital hygiene/public health measures, thus reinforcing its cost-effectiveness.

All strains were recovered from severe invasive GAS infections and thus, the analysis of the virulome was very interesting. However, the major limitation of the investigation of the genetic basis of the virulence of particular strains is the exhaustiveness of public databases. Here, we used only the curated and commonly used VFDB. Interestingly, all the isolates encoded between 2 to 5 genes homologous to known superantigens (mean = 4), but only five patients developed a toxic shock syndrome, thus supporting the evidence that the finding of CDS does not correlate with virulence [9]. Consistent to previous observations [28, 29], *emm*22 and *emm*89 isolates lacked the complete *has*-operon, although they were collected from patients with severe clinical presentation, thus emphasizing that hyaluronic acid capsule is not a prerequisite for increased virulence. Moreover, *emm*28 isolates (ISR9 and ISR11) had a premature truncation in the *hasA* product and did not produce an increased amount of hyaluronic acid despite the presence of mutations in *covS*, generally associated with increased hyaluronic acid production. In total, three mutations found in the regulatory genes *rocA* and *covS* (ISR1, ISR9 & ISR11) were previously correlated to more virulent phenotypes [24, 25]. While identifying mutations in the regulatory pathways of virulence is a promising way to predict the virulence of a given strain based on genomic data, no comprehensive database is currently available and the published data concern mainly *emm*3 and *emm*1 strains. Further research is thus required in this field.

Going deeper into virulence characterization, Olsen et al. [30] provided a response to a mock outbreak of GAS by performing WGS, genome-wide transcript analysis, and mouse virulence studies in a short time period. Here, we do not think that transcriptome analysis or *in vivo* studies were required as an outbreak of a single hypervirulent clone was already ruled out and further characterization of virulence factors would be difficult to perform and interpret due to our limited sample size with such heterogeneity of *emm*-types. Indeed, we only aimed to report findings from a real time investigation of a putative outbreak rather than conducting a study on virulence.

Concerning antibiotic resistances, the presence of antibiotic resistance genes *tet(M)* and *ermB* correlated with reduced antibiotic susceptibility. However, Resfinder did not detect point mutations in genes encoding for the antibiotic target such as *gyrA* or *parC*. Our study showed that a known mutation in *parC* conferring quinolone resistance to both *emm*22 isolates could only be found by additional specific analysis, emphasizing a lack of automated tools for the detection of point mutations in genes encoding for drug targets.

In conclusion, our study demonstrates the usefulness of whole genome sequencing by providing short time-to-results, by discriminating between closely related isolates when suspecting an outbreak but also by investigating the virulome, including its regulatory mechanisms as well as the antibiotic resistome. As such, WGS should be broadly implemented in clinical microbiology laboratories to improve patient care.

### Accession numbers

Assemblies have been deposited on the DDBJ/EMBL/Genbank database under the study accession number PRJEB14938. The new allele and ST type were submitted to the PubMLST database.

### Acknowledgements

The computations were performed at the Vital-IT (http://www.vital-it.ch) Center for high-performance computing of the SIB Swiss Institute of Bioinformatics. We would like to thank Sébastien Aeby and Maria Senra Ortiz for their significant technical help.

## Electronic supplementary material

Below is the link to the electronic supplementary material.

ESM 1 (PDF 256 kb)
