# Supplementary material for: Importance of whole genome sequencing for the assessment of outbreaks in diagnostic laboratories: analysis of a case series of invasive Streptococcus pyogenes infections
Source: Eur J Clin Microbiol Infect Dis. 2017 Jan 26;36(7):1173–80. doi: 10.1007/s10096-017-2905-z (PMC5495845; doi:10.1007/s10096-017-2905-z)
Supplement: Supplementary file 1 — (PDF 256 kb) [file 10096_2017_2905_MOESM1_ESM.pdf]

Supplementary materials

Table S1 List of the complete *Streptococcus pyogenes* genomes used for the phylogenetic analysis Core-genome phylogeny was done using Parsnp [1]. SF370 was used as reference for the detection of mutations in *covR, covS, rocA, ropB*. FDAARGOS_149, MGAS8232 and STAB10015 were used as references to detect SNPs in the core-genome of the 3 groups of closely related isolates (ISR3 & ISR4, ISR7 & ISR8 and ISR9 & ISR11, respectively).

Strain	BioSample	BioProject	Assembly	Size (Mb)	GC%	Genes	Proteins	Release Date
**SF370**	SAMN02604089	PRJNA269	GCA_000006785.2	1.85243	38.5	1801	1693	10.04.01
**MGAS8232**	SAMN02603495	PRJNA286	GCA_000007285.1	1.89502	38.5	1920	1794	31.01.02
**MGAS315**	SAMN02603496	PRJNA311	GCA_000007425.1	1.90052	38.6	1923	1804	18.07.02
**Manfredo**	SAMEA1705956	PRJNA270	GCA_000009385.1	1.84127	38.6	1841	1720	05.02.07
**SSI-1**	-	PRJNA301	GCA_000011285.1	1.89427	38.6	1912	1808	04.03.03
**MGAS10394**	SAMN02603497	PRJNA12469	GCA_000011665.1	1.89988	38.7	1875	1738	03.08.04
**MGAS5005**	SAMN02603500	PRJNA13888	GCA_000011765.2	1.83856	38.5	1835	1724	05.08.05
**MGAS6180**	SAMN02603499	PRJNA13887	GCA_000012165.1	1.89757	38.4	1870	1748	04.08.05
**MGAS9429**	SAMN02603501	PRJNA16363	GCA_000013485.1	1.83647	38.5	1800	1680	04.05.06
**MGAS10270**	SAMN02603502	PRJNA16364	GCA_000013505.1	1.92825	38.4	1923	1778	04.05.06
**MGAS2096**	SAMN02603503	PRJNA16365	GCA_000013525.1	1.86035	38.7	1809	1609	04.05.06
**MGAS10750**	SAMN02603504	PRJNA16366	GCA_000013545.1	1.93711	38.3	1924	1770	04.05.06
**NZ131**	SAMN02604236	PRJNA20707	GCA_000018125.1	1.81578	38.6	1791	1626	16.10.08
**Alab49**	SAMN02603353	PRJNA73399	GCA_000230295.1	1.82731	38.6	1816	1699	07.10.11
**MGAS15252**	SAMN02603944	PRJNA49669	GCA_000250905.1	1.75083	38.5	1693	1587	01.03.12
**MGAS1882**	SAMN02603945	PRJNA66081	GCA_000250925.1	1.78103	38.5	1728	1624	01.03.12
**A20**	SAMN02603640	PRJNA175952	GCA_000307535.1	1.83728	38.5	1833	1724	22.10.12
**M1 476**	-	PRJDB141	GCA_000349925.2	1.83113	38.5	1820	1721	14.07.12

**HSC5**	SAMN02604317	PRJNA205050	GCA_000422045.1	1.81835	38.5	1806	1686	11.07.13
**STAB902**	SAMN03081524	PRJNA231895	GCA_000732425.1	1.89212	38.5	1912	1804	22.07.14
**ATCC 19615**	SAMN02905033	PRJNA244349	GCA_000743015.1	1.8448	38.5	1835	1702	22.08.14
**M23ND**	SAMN02800743	PRJNA248257	GCA_000756485.1	1.84648	38.6	1839	1719	22.09.14
**7F7**	SAMN02644974	PRJNA238516	GCA_000767505.1	1.70979	38.6	1653	1546	20.10.14
**HKU360**	SAMN02980885	PRJNA257934	GCA_000772185.1	1.94454	38.5	1916	1809	05.11.14
**1E1**	SAMN02649402	PRJNA238987	GCA_000772245.1	1.79615	38.5	1741	1614	05.11.14
**AP1**	SAMN02716635	PRJNA242701	GCA_000993765.1	1.90829	38.5	1930	1830	07.05.15
**JRS4 (Wash.)**	SAMN03610013	PRJNA283215	GCA_001014285.1	1.81197	38.6	1788	1667	28.05.15
**D471**	SAMN03610015	PRJNA283214	GCA_001014305.1	1.81197	38.6	1788	1667	28.05.15
**NGAS743**	SAMN02715744	PRJNA243328	GCA_001019635.1	1.91555	38.5	1924	1805	03.06.15
**NGAS596**	SAMN02715758	PRJNA243328	GCA_001019675.1	1.79131	38.5	1739	1617	03.06.15
**NGAS327**	SAMN02715759	PRJNA243328	GCA_001019695.1	1.70205	38.6	1644	1537	03.06.15
**M28PF1**	SAMN03733610	PRJNA284654	GCA_001020185.2	1.89698	38.4	1874	1752	05.06.15
**5448**	SAMN02866032	PRJNA252999	GCA_001021955.1	1.82952	38.5	1817	1725	08.06.15
**STAB10015**	SAMN03418291	PRJNA278400	GCA_001023495.1	1.95045	38.2	1917	1800	09.06.15
**H293**	SAMEA3865279	PRJEB1935	GCA_001039695.2	1.72625	38.6	1661	1539	13.05.14
**HKU488**	SAMN03846812	PRJNA289181	GCA_001051095.1	1.94341	38.5	1944	1832	09.07.15
**NGAS322**	SAMN03274509	PRJNA243328	GCA_001267805.1	1.95047	38.3	1929	1809	17.08.15
**NGAS638**	SAMN03274510	PRJNA243328	GCA_001267845.1	1.7914	38.6	1758	1650	17.08.15
**MEW427**	SAMN04419118	PRJNA308988	GCA_001535505.1	1.81445	38.5	1767	1554	25.01.16
**MEW123**	SAMN04419117	PRJNA308987	GCA_001535565.1	1.8787	38.3	1827	1653	25.01.16
**JRS4 (Tokyo)**	-	PRJDB114	GCA_001547715.1	1.81112	38.6	1785	1657	04.04.15
**MTB313**	SAMD00000328	PRJDB1654	GCA_001547815.1	1.74533	38.5	1843	1758	24.02.15
**MTB314**	SAMD00000332	PRJDB1668	GCA_001547835.1	1.74483	38.5	1722	1588	24.02.15
**FDAARGOS_149**	SAMN03996295	PRJNA231221	GCA_001559175.1	1.83964	38.5	1831	1720	11.02.16
**AP53**	SAMN04334485	PRJNA305806	GCA_001620285.1	1.86055	38.6	1862	1742	18.04.16
**MGAS11027**	SAMN03792044	PRJNA167314	GCA_001635895.1	1.78687	38.6	1738	1617	02.05.16
**MGAS23530**	SAMN03775331	PRJNA287028	GCA_001635935.1	1.70939	38.5	1640	1527	02.05.16

**MGAS27061**	SAMN03161558	PRJNA266333	GCA_001635975.1	1.74135	38.5	1675	1565	02.05.16
**NS53**	SAMN04625972	PRJNA317786	GCA_001638845.1	1.76512	38.4	1693	1587	06.05.16
**STAB09014**	SAMN03418290	PRJNA278400	GCA_001678885.1	1.86249	38.4	1823	1697	05.07.16
**STAB901**	SAMN03081523	PRJNA232121	GCA_000732385.1	1.79561	38.5	1812	1358	22.07.14

Table S2 Assemblies statistics determined using Quast version 3.1 [2], median coverage determined by mapping the trimmed reads on the assemblies and accession numbers

	ISR1	ISR2	ISR3	ISR4	ISR5	ISR6	ISR7	ISR8	ISR9	ISR10	ISR11
**Number of contigs (>= 0 bp)**	71	32	58	35	73	36	41	53	22	19	32
**Number of contigs (>= 1000 bp)**	29	19	23	17	30	26	26	33	18	16	21
**Number of contigs (>= 10000 bp)**	14	12	15	13	18	19	18	19	12	12	15
**Number of contigs (>= 50000 bp)**	12	12	11	10	11	11	13	13	10	10	10
**Largest contig**	516565	305309	751698	751702	278987	265863	251794	251794	504539	630053	529116
**Total length (>= 0 bp)**	1838723	1866975	1820544	1809116	1863500	1856594	1849575	1877415	1823476	1690061	1850892
**Total length (>= 1000 bp)**	1823729	1862425	1804961	1802965	1846068	1852149	1844325	1868156	1822707	1689494	1847894
**Total length (>= 10000 bp)**	1791557	1847827	1788337	1793215	1817829	1839380	1822960	1812159	1809795	1680544	1834982
**Total length (>= 50000 bp)**	1729935	1847827	1688855	1732523	1649245	1633436	1641065	1598590	1759057	1604391	1702270
**N50**	171252	225304	203845	221880	219677	206099	133991	134362	243478	163042	243478
**L50**	4	4	2	2	4	4	5	5	3	3	3
**GC (%)**	38.42	38.48	38.36	38.39	38.38	38.43	38.34	38.33	38.19	38.4	38.21
**Median coverage**	430	349	270	372	150	205	184	203	196	216	173
**Contigs accession number**	ERS 1420141	ERS 1420142	ERS 1420143	ERS 1420144	ERS 1420145	ERS 1420146	ERS 1420147	ERS 1420148	ERS 1420149	ERS 1420150	ERS 1420151

Table S3 Mutations detected in 4 different regulatory genes (*covR, covS, rocA, ropB*) and their effect on gene products
WT: Wild Type, nr: nucleotide replacement, del: deletion.
*mutations described by Ikebe et al. [3] °mutations described by Lynskey et al. [4]

Strain	*covR*	Effect	*covS*	Effect	*rocA*	Effect	*ropB/rgg*	Effect
emm 1								
ISR3	WT	-	nr	I332V	WT	-	WT	-
ISR4	WT	-	nr	I332V	WT	-	WT	-
emm 22								
ISR7	Nr	V128A	nr	-	nr	R259Q, V333A	WT	-
ISR8	Nr	V128A	nr	-	nr	R259Q, V333A	WT	-
emm 28								
ISR9	Nr	-	nr	E226G*	nr	R259Q, D396N, I404T	nr	-
ISR11	nr	-	Del 1bp (base 508)	Truncation at aa. 180*	nr	R259Q, D396N, I404T	nr	-
Other *emm*-types								
ISR1 - *emm* 3	Nr	-	nr	-	nr, 1bp del (base 1228)	I203N, L247F, R259Q, D396N, I404T, truncation at aa. 416°	nr	-
ISR2 - *emm* 75	WT	-	nr	S337L	nr	R259Q	nr	-
ISR5 - *emm* 49	Nr	-	nr	-	nr	R259Q	nr	-
ISR6 - *emm* 5	Nr	-	nr	K498N	nr	V47A, R259Q, D396N, I404T, P405S	nr	V169I
ISR10 - *emm* 89	Nr	-	nr	-	nr	R259Q	nr	-

Table S4 Virulence factor genes their products, extracted from the Virulence Factor Database [5]

Virulence factors	
ccmA : ABC transporter involved in cytochrome c biogenesis, ATPase component CcmA	*mf/spd* : Streptodornase
cheA : chemotaxis histidine kinase	*mf2* : deoxyribonuclease, phage-associated
cpa : fibronectin-binding protein, GAS pilus tip adhesin	*mf3* : deoxyribonuclease
EF0818 : polysaccharide lyase, family 8 (Hyaluronidase)	*scpA* : C5a peptidase precursor
emm : M protein	*scpB* : streptococcal C5a peptidase
fbaA : protein F2 like fibronectin-binding protein	*sic* : streptococcal inhibitor of complement
fbaB : fibronectin-bing protein SfbII	*ska* : streptokinase A precursor
fbp54 : fibronectin-bing protein Fbp54	*slo* : streptolysin O precursor
fctA : major pilin FctA	*smeZ* : streptococcal mitogenic exotoxin Z
fctB : minor pilin FctB	*spea* : streptococcal exotoxin A precursor, phage-associated
grab : protein G-related alpha 2M-bindingprotein	*speB* : pyrogenic exotoxin B
hasA : hyaluronate synthase	*spec* : streptococcal exotoxin C precursor, phage-associated
hasB : UDP-glucose 6-dehydrogenase	*speg* : streptococcal exotoxin G precursor
hasC : UDP-glucose pyrophosphorylase	*speh* : streptococcal exotoxin H precursor
hylP : hyaluronidase, phage-associated	*spei* : streptococcal exotoxin I precursor
ideS/mac : immunoglobulin G-degrading enzyme	*spej* : streptococcal exotoxin J precursor
lepA : signal peptidase I	*spek* : streptococcal exotoxin SpeK, phage-associated
lmb : Laminin-binding surface protein	*srtC1* : sortase

1. Treangen TJ, Ondov BD, Koren S, Phillippy AM (2014) The Harvest suite for rapid core-genome alignment and visualization of thousands of intraspecific microbial genomes. Genome Biol 15:524. doi: 10.1186/PREACCEPT-2573980311437212

2. Gurevich A, Saveliev V, Vyahhi N, Tesler G (2013) QUAST: quality assessment tool for genome assemblies. Bioinforma Oxf Engl 29:1072–1075. doi: 10.1093/bioinformatics/btt086

3. Ikebe T, Ato M, Matsumura T, et al (2010) Highly frequent mutations in negative regulators of multiple virulence genes in group A streptococcal toxic shock syndrome isolates. PLoS Pathog 6:e1000832. doi: 10.1371/journal.ppat.1000832

4. Lynskey NN, Turner CE, Heng LS, Sriskandan S (2015) A Truncation in the Regulator RocA Underlies Heightened Capsule Expression in Serotype M3 Group A Streptococci. Infect Immun 83:1732–1733. doi: 10.1128/IAI.02892-14

5. Chen L, Zheng D, Liu B, et al (2016) VFDB 2016: hierarchical and refined dataset for big data analysis—10 years on. Nucleic Acids Res 44:D694–D697. doi: 10.1093/nar/gkv1239
